# Informing HIV prevention efforts targeting Liberian youth: a study using the PLACE method in Liberia

**DOI:** 10.1186/1742-4755-10-54

**Published:** 2013-10-09

**Authors:** Donna R McCarraher, Mario Chen, Sam Wambugu, Steve Sortijas, Stacey Succop, Bolatito Aiyengba, Chinelo C Okigbo, Allison Pack

**Affiliations:** 1 Social and Behavioral Health Sciences, FHI 360, Durham, NC 27705, USA; 2 Biostatistics, FHI 360, Research Triangle Park, Durham, NC, USA; 3 Monitoring and Evaluation, FHI 360, Accra, Ghana, Africa; 4 Monitoring and Evaluation, FHI 360, Abuja, Abuja, Africa; 5 Gillings School of Global Public Health, University of North Carolina, Chapel Hill, NC, USA

**Keywords:** HIV prevention, Youth, Research method, Liberia, Africa

## Abstract

**Background:**

Preventing HIV infection among young people is a priority for the Liberian government. Data on the young people in Liberia are scarce but needed to guide HIV programming efforts.

**Methods:**

We used the Priorities for Local AIDS Control Efforts (PLACE) method to gather information on risk behaviors that young people (ages 14 to 24) engage in or are exposed to that increase their vulnerability for HIV infection. Community informants identified 240 unique venues of which 150 were visited and verified by research staff. 89 of the 150 venues comprised our sampling frame and 571 females and 548 males were interviewed in 50 venues using a behavioral survey.

**Results:**

Ninety-one percent of females and 86% of males reported being sexually active. 56% of females and 47% of males reported they initiated sexual activity before the age of 15. Among the sexually active females, 71% reported they had received money or a gift for sex and 56% of males reported they had given money or goods for sex. 20% of females and 6% males reported that their first sexual encounter was forced and 15% of females and 6% of males reported they had been forced to have sex in the past year. Multiple partnerships were common among both sexes with 81% females and 76% males reporting one or more sex partners in the past four weeks. Less than 1% reported having experiences with injecting drugs and only 1% of males reporting have sex with men. While knowledge of HIV/AIDS was high, prevention behaviors including HIV testing and condom use were low.

**Conclusion:**

Youth-focused HIV efforts in Liberia need to address transactional sex and multiple and concurrent partnerships. HIV prevention interventions should include efforts to meet the economic needs of youth.

## Introduction

With the support of international donor agencies, the Liberian Government is in the process of reestablishing basic services to ensure the health and well-being of its people which were dismantled as a result of a two civil wars that spanned from 1989 to 2003 [1]. HIV care and treatment are included in a basic package of health services the government is attempting to reestablish in all parts of the country [2]. The 2007 Liberian Demographic and health Survey (LDHS) reported that the HIV nationwide HIV prevalence nationwide was 1.5% (95% CI 1.3%-1.8%). The HIV prevalence was found to be higher in urban areas than rural areas, and higher among females than males. The rates of infection were three times higher among adolescent females (ages 15–19) compared males (1.3% vs. 0.4%) and almost three times higher among young females (ages 19–24) compared to young males (2.0% vs. 0.7%)[3]. Given that more than half of the population in Liberia is young, preventing HIV infection among them is critical. The data on the factors that enhance young people’s risk of getting HIV are scarce and the available evidence suggests that a milieu of structural and individual risk factors have the potential to increase youth’s vulnerability to HIV.

The structural drivers include poor access to education, high rates of gender-based violence (GBV), and limitations of the current health system. The disruption of the education system has resulted in high rates of illiteracy. In 2007, 41% of girls ages 15–19 and 47% of women 20–24 cannot read at all. Lower rates were reported among males in the same age groups, with 27% and 19% illiteracy, respectively [3]. Moreover, while efforts are underway to reestablish the educational system, many youth are still not in school. The inability to read and poor of school attendance hampers HIV efforts among young people as it limits their ability to comprehend information related to HIV prevention and eliminates an important means of reaching young people; through the schools they attend.

GBV affects HIV transmission and mitigation through numerous pathways. It has been found to be associated with HIV acquisition [4,5], lack of condom use [6-11], willingness to disclose HIV test results [12-14], and suboptimal adherence to antiretroviral therapies for treatment [15,16]. GBV is widespread in Liberia. The 2007 LDHS report that 13% of females ages 15–24 have experienced sexual violence; and 39% of female adolescents ages 15–19 report they have been victims of physical violence [3]. The United States Agency for International Development (USAID) Liberian Youth Fragility Assessment reported that half of all rape cases against girls occur with girls between the ages of 10–15 [17].

Finally, Liberia’s fragmented and under-resourced health care system has thwarted an effective response to the HIV epidemic. The Liberia 2010–2014 National HIV/AIDS Strategic Framework II reports only 89 HIV testing facilities in the country [1]. The lack of testing facilities, contributes to the low rates of HIV testing among youth; the 2007 LDHS reported that 4.4% of young women and 3.4% of young men aged 15–24 had ever been tested for HIV [3]. The lack of systematic health care system has resulted in heavy reliance on traditional healers which have not been actively engaged in HIV prevention efforts[18].

Many Liberian youth engage in individual behaviors that heighten their risk for HIV, including transactional sex resulting in multiple and concurrent partnerships, and low levels of condom use. Four studies report that conflict-related displacement has led young people to exchange sex for food and housing in order to survive and that in some cases this behavior encouraged by their parents or other adult [19-21]. The 2007 LDHS reported that 10% of 15–24 year olds who are married or living in union reported using a condom at last sex [3]. The 2010 UNAIDS Global Report revealed that among Liberian youth who had sex with a non-marital and non-cohabiting partner in the last 12 months, that 22% of males and 14% of females reported condom use at last sex [22].

In 2010, the United Nations Child Fund (UNICEF) formed a stakeholder working group with the goal of meeting the HIV prevention needs of young people in three counties in Liberia: Montserrado, Grand Gedeh, and Grand Bassa. The stakeholder working group included individuals from several international and domestic agencies. In collaboration with UNICEF and the Liberia Institute of Statistics and Geo-Information Services (LISGIS), we used the Priorities for Local AIDS Control Efforts (PLACE) method [23,24] to gather information to inform HIV youth prevention programming in these counties. PLACE is a rapid assessment tool that has been used in several countries world-wide just for this purpose. We report here on the main PLACE indicators, stratified by sex, collected among young people living in Montserrado County, which contains the capital city of Monrovia. Data from Grand Gedeh and Grand Bassa are not presented as the data collection efforts in these two counties were completed at a later date.

## Methods

The PLACE method has five steps: 1) formation of a stakeholder working group and identification of prevention priority areas; 2) community informant interviews to identify venues where people meet and engage in behaviors that place them risk of HIV; 3) verification of venues identified by community informants; 4) selection of venues to conduct data collection efforts; 5) behavior surveys conducted at selected venues.

First, in March 2011, the stakeholder working group identified Montserrado County as one target area because it is the largest county in the country, more than half of the entire population lives in it, and it has the highest HIV prevalence in the country which is 2.6%.

Subsequently, the research assistants returned to venues to ensure that 1) the venues existed and were operational; 2) young people ages 14–24 were present; and 3) they were locations where youth meet sexual partners. At each venue, research assistants interviewed a person present who was knowledgeable about the venue to obtain information about when the venue was the busiest and information on those who come to the venue. The number of young people present at each site was also noted on the venue verification questionnaire and transferred to the excel spreadsheet.

We selected a random sample of venues to include in the youth behavioral survey using a selection with probability proportional to the size (PPS) of the venue. The number of people socializing at the venue as recorded during mapping activities was used as measure of size for PPS sampling. We ordered the sampling frame of venues by zone and size for an implicit stratification and selected the venues using a systematic approach. This approach should produce an approximately self-weighted sample in which each venue patron had a similar selection probability to be included in the sample. Sixty clusters of size 16 – for a total target recruitment of 960 individuals 14 to 25 years old – were selected among the venues for the behavioral survey. The sample size of 960 is usually recommended for PLACE studies to allow for the estimation of key indicators with adequate precision. This sample size accounted for the design effect due to the clustered sampling design. We selected more clusters and fewer participants per clusters than the combination of 45 clusters with 24 participants more commonly used with PLACE because the number of small venues identified. The 60 clusters selected corresponded to 45 venues since some large venues contained several selected clusters. Additional five clusters were selected to reach the target sample size when interviews could not be completed in some venues during the field work.

Research assistants returned to selected venues to conduct the youth behavioral survey. We instructed the research assistants to identify and conduct interviews with young people between the ages of 14 and 25 at the selected venues who were not accompanied by a parent or on a family errand. Venues were visited in teams of two, and most venues were visited over two days during busy times to reach the targeted sample size at each venue. When more than one cluster was selected in a venue, more field teams were assigned to the venue in order to achieve the expected number of interviews completed per team member. No strict instructions were given to select specific numbers of males and females or how to select individuals at the venue, but interviewers were told to recruit similar numbers of males and females unless unless a venue was mostly attended by one gender.

We had intended that the behavioral survey data to be collected using personal digital assistants (PDAs) to ensure rapid data collection and to expedite the data cleaning process. The research assistants did not adapt well to the new technology and some feared that actually having the PDAs could make them targets of theft. Therefore, the behavioral surveys were conducted on hard copies and the PDAs were used for data entry at the LISGIS office in Monrovia. The data were usually entered the day after the survey was completed. The information from the PDAs was downloaded onto laptops, verified for accuracy, and transferred to the FHI 360 North Carolina office via e-mail for analysis on a regular basis during the field work. The complete datasets for each data collection phase were converted into SAS (9.3) for analysis.

Behavioral surveys collected information for key indicators on sexual behaviour, drug use, men who have sex with men, condom use, and HIV counselling and testing experiences among others. Data collection was initiated in October/November 2011, suspended due to local elections, and reinitiated and completed in January 2012.

### Data analysis

Results from the venue verification interviews and behavioral survey are presented descriptively. We did not account for clustering by venue because our approach was to achieve a self-weighted sample. Results from the behavioral survey are stratified by gender. We provide 95% confidence intervals for key indicators of interest. These confidence intervals account for the design effect due to the selection of venues and participants within venues.

## Results

The community informant interviews led to the identification of 240 venues. During the verification process, we discovered that 23 venues were closed, 42 did not exist, 16 existed but did not have young people present at them, and 9 venues were duplicates. This resulted in 150 unique venues that were operational and had young people present at them. However, the sampling frame for this analysis was drawn from 89 of the 150 verified venues because we drew the sample from a spreadsheet compiled in the field as the venue verification process was on-going which did not have all 150 venues entered into it. Subsequently, we received the database that contained the data from the venue verification forms and determined that 150 venues had been visited and verified.

For comparison purposes, information about the venues verified, those that comprised the sampling frame, and the venues in which interviews were conducted are displayed in Table 1. There were no major differences across the three groups. The majority of these venues were places where individuals eat, drink, or sleep. In descending order, these venues included nightclubs, bars, hotels/motels, restaurants, and informal bars. The transportation/public/commercial areas included video stores, liquor stores, street corners, beaches, and taxi stands. The Hidden/Private/Abandoned Areas consisted of private homes within poor areas. Finally, music shows were the only events mentioned.

**Table 1:** Information on venues verified, eligible venues, and venues in which surveys were conducted

	**Verified N=150 N (%)**	**Sampling Frame N=89 N (%)**	**Surveys Conducted N=50 N (%)**
Venue Type			
Eating/Drinking/Sleeping Venues	122 (81.3)	68 (76.4)	39 (78.0)
Transportation/public/commercial areas	15 (10)	9 (10.1)	2 (4.0)
Hidden/Private/Abandoned Areas	11 (7.3)	10 (11.2)	8 (16.0)
Events	2 (1.4)	2 (2.3)	1 (2.0)
Position of individual interviewed during venue verification	N=142	N=86	N=48
Owner/manager	42 (29.6)	20 (23.3)	11 (22.9)
Staff member	42 (29.6)	23 (26.7)	13 (27.1)
Patron	17 (12.0)	12 (13.9)	6 (12.5)
Missing	41 (28.8)	31 (36.1)	18 (37.5)
Proportion of individuals interviewed reporting that females (ages 14-25) engage in the following activities at venues			
Drink Alcohol	117 (82.4)	74 (86.1)	44 (91.7)
Find new sex partner	77 (54.2)	63 (73.3)	38 (79.2)
Appear to be injection drug users	7 (4.9)	7 (8.1)	4 (8.3)
Appear to be selling or buying sex	48 (33.8)	39 (45.4)	23 (47.9)
Proportion of individuals interviewed reporting that males (ages 14-25) engage in the following activities at venues			
Drink Alcohol	121 (85.2)	76 (88.4)	45 (93.7)
Find new sex partner	72 (50.7)	60 (69.8)	38 (79.2)
Appear to be injection drug users	11 (7.8)	10 (11.6)	5 (10.4)
Appear to be selling or buying sex	42 (29.6)	36 (41.9)	25 (52.1)
Men have sex with men	10 (7.0)	9 (10.5)	5 (10.4)
HIV Prevention activities reported at venues			
HIV/AIDS radio broadcast	77 (54.2)	45 (52.3)	28 (58.3)
Condom promotion	45 (31.7)	28 (32.6)	14 (29.2)
HIV posters/leaflets	33 (23.2)	20 (23.3)	11 (22.9)
Peer health educator program	31 (21.8)	18 (20.9)	11 (22.9)
Condoms currently either free or sold at venue	42 (29.6)	25 (29.1)	15 (31.25)

Eight individuals approached at the venues for a venue verification interview did not consent to be interviewed. Half of the individuals interviewed at the venues where the behavioral survey was conducted were owners or individuals who worked at the venue in some capacity. Almost all of the individuals interviewed reported that young men and women came to the venue to drink alcohol; about 80% reported they came to meet new sexual partners, and about 50% reported young people were either buying or selling sex at the venue. Ten percent or less reported that injection drug use occurred at the venue or that the venue was a location where men were seeking sex with other men. A variety of HIV prevention activities were reported at the venues with radio programs being the most commonly reported (58%). A third reported that condoms were available at the venue for free or sold at the venue.

### Behavioral survey

A total of 186 interviews were conducted in September/October 2011. A total 135 interviews from the first period were discarded from research assistants who exhibited unprofessional conduct, mainly drinking at the study venues which made them unable to properly administer the surveys. The misconduct was reported by the study supervisors employed by LISGIS. We decided to discard all the interviews conducted by these staff members. Only research assistants who were considered to have adequately followed field procedures during the first period were retrained and allowed to participate in the second interview period. A total of 1068 interviews were conducted in December 2011-January 2012. The total sample of this analysis is 1119 interviews (571 females and 548 males).

### Socio-demographic information

Forty-two percent of the males interviewed and 51.6% of the females interviewed were adolescents (Table 2). About one quarter of them reported they lived alone or on the street. 83.7% of males and 73.7% of females had completed a primary school education or more. A quarter of males and half of the females reported that they were unemployed. Almost all study participants were from Montserrado County. Over half of the females interviewed reported they had been pregnant previously and 5.4% reported they were currently pregnant.

**Table 2:** Socio-demographic characteristics

	**Males n=571**	**Females n=548**
	**(%)**	**(%)**
Age		
14-19 years	42.0	51.6
20-25 years	58.0	48.4
Lives alone or on street	26.4	22.8
Education		
None	3.0	8.8
Some Primary	12.8	16.6
Completed Primary or higher	83.7	73.7
Missing	0.5	0.9
Unemployed	25.2	49.8
Lives in PPA	98.9	98.7
Ever pregnant	xx	53.7
Currently pregnant	xx	5.4

### Sexual behavior

86% of males and 91.1% of females reported they were sexually active (Table 3). The mean age of first sex for males was 15.7 years and was 15.2 years for females. Few study participants reported that their first sexual partner was 10 years older than them or more. Almost 20% of females reported their first sexual encounter was forced. 15.0% of females and 6.0% of males reported that they had been forced to have sex in the past year (data not shown) Over half the males interviewed reported they had given money or other items for sex, and 71.1% of the females reported they had received money or other items for sex. The vast majority received or gave money for sex (>90%) and few reported receiving shelter, food, or school fees (data not shown).

**Table 3:** Sexual behavior characteristics

	**Males n=491**		**Females n=499**	
	**(%)**	**95%CI**	**(%)**	**95% CI**
Sexually active	86.0	83.1-88.8	91.1	88.7-93.5
Age at first sex				
<14	13.0		19.2	
14-29	83.3		80.0	
20-25	3.7		0.8	
Mean	15.7		15.2	
First sex partner 10 years or older	5.9		11.2	
First sex forced	5.9		19.6	
Ever given money for sex	56.2		8.4	
Ever received money for sex	12.8		71.1	
Sexual Activity last 4 weeks				
Had new sex partner in last four weeks	73.9	70.0-77.8	83.2	79.9-86.5
More than 1 sex partner in last 4 weeks	76.4	72.6-80.1	81.4	77.9-84.8
Of these, used condom last sex act	44.4	40.0-48.8	50.9	46.5-55.3
Sex Rate Among All^1^				
High (≥1 new or >2 partners in last 4 weeks)	71.5	67.7-75.2	79.6	76.2-82.9
Moderate (1 new or ≥2 partners in last 12 months)	9.6	7.2-12.1	7.8	5.6-10.1
Low (≤1 partner in last 12 months)	4.9	3.1-6.7	3.6	2.1-5.2
No Sex	12.4	9.7-15.1	5.8	3.9-7.8
Used Condom last sex	56.2	1.8-60.6	59.1	54.8-63.4
Men ever had sex with men^2^	1.2		xx	
Last sex partner 10 years or older	4.3		20.4	
Ever pregnant	xx		53.7	
Currently pregnant	xx		5.4	

^1^ = n = 571 males and n = 548 females; ^2^n = 571 males.

The majority of study participants reported they had a new sex partner in the past 4 weeks (73.9% of males and 83.2% of females) and three-quarters or more reported they had more than one sexual partner in the past four weeks. Condom use at last sex act among those who reported sexual activity in the past four weeks was low (less than 50%) among males and females. Rates of new sexual partner acquisition were high for both males (71.5%) and females (79.6%). Only 1.2% of males reported they have ever had sex with men.

### Alcohol and drug use

Few study participants reported they had ever injected drugs (Table 4). However, 25.9% of males and 21.5% of females reported they had tried hard drugs (defined as cocaine, opium, heroin, marijuana, or valium) at some point. Over three-quarters of study participants reported they used alcohol in the four weeks prior to the survey, and 30% of males and females reported that they drank daily in the past four weeks.

**Table 4:** Drug use and alcohol use

	**Males 571**		**Females 548**	
	**(%)**	**95% CI**	**(%)**	
Ever injected drugs	0.9	0.1-1.6	0.4	0.0-1.4
Injected drugs past four weeks	0.4		0.2	
Ever use hard drugs^1^	25.9		21.5	
Ever used alcohol	75		77	
Used alcohol every day in past 4 weeks^2^	30		30	

^1^ = Cocaine, Heroin, Marijuana, Valium, Opium, or other.

^2^ = Among those who report alcohol use n = 432 males; n = 419 females.

### HIV knowledge and prevention

The majority of the study participants reported they had heard of HIV (Table 5). The main form in which study participants had received information about HIV was through radio programs (55% males and 54% females). Only 9.9% of males reported they had spoken to a health worker about HIV. 15.2% of males and 21.4% of females reported they got an HIV test. Among those who were tested, the majority reported they got their results. Slightly more than half of the study participants reported they knew where they could get an HIV test. Most participants reported that HIV testing was available at hospitals with few reported other options such as standalone HIV testing centers or mobile units (data not show).

**Table 5:** HIV knowledge, education and testing

	**Males n = 571**		**Females n = 548**	
	**%**	**95% CI**	**%**	**95% CI**
Have heard of HIV	95.8	94.1-97.4	94.0	92.0-96.0
Exposure to HIV education in past 12 months				
Heard program about HIV on radio	55.0	12.2-18.2	54.0	
Attended HIV education program	13.7		10.7	
Seen HIV video	13.0		10.5	
Talked about HIV with health worker	9.9		17.1	
Ever had HIV test	15.2		21.4	17.8-24.9
Got HIV test result^1^	92.8		90.0	
Knows a place to get an HIV test	56.7		59.4	

^1^ = n = 83 males and 99 females.

## Discussion

Using the PLACE methodology, we found that multiple partnerships, the exchange of sex for money, and low levels of use of condoms were the main HIV risk factors exhibited by those we interviewed. Very few individuals reported injection drug use and only 1% of men reported have sex with other men. While knowledge about HIV was high, only a minority of study participants reported engaging in preventative measures. Condom use was low among study participants and HIV testing was poorly utilized. These findings reinforce the policy recommendation put forth by the National AIDS Commission in Liberia, namely that addressing multiple and concurrent partnerships and transactional sex need to be at the core of HIV prevention efforts for young people.

Our use of the PLACE method resulted in identifying young people at risk of HIV. Comparing our study population to the 2007 LDHS, we found higher rates multiple partnerships (over 70% reported one or more new partners or two or more partners in the past four weeks) as compared to 2007 LDHS (less than 12% reported two or more partners in the last 12 months). Compared to the DHS, we found higher rates of forced sex at first sexual encounters among females (almost 20%) compared to the LDHS (<14%). We found similar rates of HIV testing between our sample and the LDHS. However, we found condom use among the young people we interviewed to be higher than that reported by the LDHS [3] and other sources [25].

There are several limitations to this study. First, because the key informant interview transcripts were lost in shipping we lack information on the community informants that would have shed light on those who identified the venues initially. Second, our sample frame of venues was a subset of all venues verified. While are comparisons revealed no differences in between the verified venues, our sampling frame, and the venues in which interviews were ultimately conducted, there might some selection bias associated with factors that we did not measure. There was variation between the spreadsheet and the final database that contained the venue verification forms because the research assistants were allocated to different areas and streets to verify the venues they were assigned. In many cases, inadequate information about the venues led to a lot of time being spent locating venues. In addition, budget and time constraints made it impossible to allow time between the venue verification process and implementing the behavioral survey. Third, the data cleaning process revealed several consistency problems in the data, calling into question the readiness of the interviewers to conduct the surveys and ultimately the quality of the data. A decision was made to report conservative estimates (data we coded to bias results towards the null) as a means of addressing this problem. Finally, we were unable to gain insight into the behaviors of MSM or IDUs. This could be because venues where young people meet new sex partners were highlighted during interviewer training. In addition, we did attempt to over sample venues where these groups can be found.

Despite these limitations, we consider the PLACE method to be fairly simple method to gather important data on difficult topics in a rapid manner. After the termination of some research assistants suspected of misconduct, the remaining research assistants conducted the surveys professionally and efficiently and survey participants were receptive of the survey. They managed to approach potential participants and complete the interviews under challenging conditions (e.g., low light and loud noise), and there were no indications –as reported by field supervisors- that patrons felt threatened or uncomfortable because of the presence of the field team. We believe the data collected provide a realistic picture of the issues affecting youth in Montserrado as they relate to HIV risk behaviors, but decisions based on these data should be made with caution as generalizability to the larger population is limited.

We presented the results of this study to the stakeholder working group and others at a day long workshop in December 2012. In addition, we presented the group with a host of intervention strategies that have been proven to help increase knowledge about HIV, change HIV-related risk behaviors, and improve sexual and reproductive health outcomes among youth. These included youth- friendly services, economic empowerment, sexuality education, peer education, cash transfers, and broad community engagement. Meeting attendees determined that each of these strategies should be utilized in Liberia and the stakeholders, working in small groups, discussed how to domesticate each strategy for the Liberian context. All stakeholder recommendations for domestication were presented by strategy area and included: 1) target behaviors, such as transactional sex and condom use, to highlight in the strategy; 2) key program details, such as the steps necessary to enact the selected strategy; 3) potential collaborating agencies; 4) notes on the feasibility of the strategy; and 5) a description of linkages between the selected strategy and others strategies. Information from this meeting was summarized in a youth HIV prevention strategy for UNICEF and other funders, decision-makers and program planners in Liberia who are responsible for current and future programs for young people [26].

Future research should include a qualitative study in the areas where this work was conducted to highlight some of the contextual factors that explain why youth engage in behaviors that place them at risk of HIV and why they do not seem to avail themselves of the preventions strategies available. Information on IDUs and MSM is still needed. This information could be obtained by using the PLACE method targeting venues where these sub-groups of youth congregate or another option could be to use respondent driven sampling.

## Abbreviations

AIDS: Acquired Immune Deficiency Syndrome; CI: Confidence Interval; GBV: Gender-based Violence; HIV: Human Immunodeficiency Virus; IDU: Intravenous Drug Users; LDHS: Liberian Demographic and Health Survey; LISGIS: Liberia Institute of Statistics and Geo-Information Services; MSM: Men who have Sex with Men; PDA: Personal Digital Assistants; PHSC: Protection of Human Subjects Committee; PLACE: Priorities for Local AIDS Control Efforts; PPS: Probability Proportional to Size; UNICEF: United Nations Child Fund (formerly United Nations International Children’s Emergency Fund); USAID: Unites States Agency for International Development.

## Competing interests

The authors declare that they have no competing interests.

## Authors’ contributions

DRM designed the study, wrote the first draft of document, and modified the finalized the manuscript based on comments from co-authors and referees. MC was the lead statistician on the study, reviewed and contributed to the study protocol, oversaw the study sampling, and contributed significantly to the development of the first draft of the methods section. SW, SSortijas, and BA worked with LISGIS to implement the study, monitored the data collection activities, and provided input to the manuscript. SSuccop, CO, and AP supported the implementation of the study, conducted data analysis, and provided input to the manuscript. All authors read and approved the final manuscript.
